# The effect of Licochalcone A on proliferation, invasion, and drug resistance of glioma cells by regulating TLR4/NF-κB signaling pathway

**DOI:** 10.1016/j.clinsp.2024.100542

**Published:** 2024-12-20

**Authors:** Baigui Zhou, Kun Mu, Xuzhou Yu, Xu Chen, Xiaoying Shi

**Affiliations:** a Department of Neurology, Zhejiang Jinhua Guangfu Tumor Hospital, Zhejiang, PR China; b Department of Respiratory Oncology, Zhejiang Jinhua Guangfu Tumor Hospital, Zhejiang, PR China; c Department of Emergency, Zhejiang Jinhua Guangfu Tumor Hospital, Zhejiang, PR China

**Keywords:** LCA, Glioma of the brain, Toll like receptor-4, Nuclear factor κB, Cell proliferation, Drug resistance

## Abstract

**Objective:**

Based on Toll Like Receptor 4 (TLR4)/Nuclear Factor-κB (NF-κB) Exploring the effects of Licochalcone A (LCA) on the proliferation, invasion, and drug resistance of glioma cells through signaling pathways.

**Methods:**

Cultivate human glioma cell line U251 in vitro, induce drug-resistant cell line U251/TMZ with Temozolomide (TMZ), and validate the results. Different concentrations of licorice chalcone A were used to treat U251 cells and U251/TMZ cells, and were named as control group, low-dose group, medium-dose group, and high-dose group, respectively. CCK-8 assay, cell adhesion assay, and Transwell assay were used to detect cell survival rate, cell adhesion rate, number of migrating cells, and number of invading cells, respectively.

**Results:**

The cell survival rate, cell adhesion rate, number of migrating and invading cells in the high-dose group were lower than those in the medium-dose group and lower than those in the control group. High-dose group TLR4, NF-κB mRNA and protein levels were lower than those in the medium dose group and lower than those in the control group (*p* < 0.05). Compared with the si-NC group, the si-TLR4 group showed a decrease in cell survival rate and adhesion rate, as well as a decrease in the number of migrating and invading cells, the levels of CyclinD1 and N-cadherin proteins decreased, while the levels of E-cadherin protein increased (*p* < 0.05).

**Conclusion:**

LCA could inhibit the proliferation and metastasis of glioma cells and reverse drug resistance, possibly by inhibiting the TLR4/NF-κB signaling pathway.

## Highlights

• LCA has the ability to suppress the growth, movement, and invasion of glioma cells.
• It can also counteract resistance to TMZ drugs.
• This opens up new possibilities for identifying clinical drugs to treat glioma patients who are resistant to TMZ.

## Introduction

Glioma is a prevalent primary tumor that occurs within the brain, accounting for almost 30 % of all malignancies in the central nervous system.^1^ The 5-year survival rate is roughly 9.8 %, indicating a significant level of malignancy. Clinically, surgical resection, chemoradiotherapy, and other treatments are commonly employed. However, prolonged chemotherapy can result in undesirable side effects. Temozolomide (TMZ) is commonly used as an initial therapy for gliomas. However, it is prone to inducing drug resistance in tumors, which limits its effectiveness in clinical practice. Therefore, it is imperative to investigate alternative treatment strategies to overcome TMZ drug resistance. Hence, it is imperative to explore innovative therapeutic strategies to overcome resistance to TMZ.^2^ Licochalcone A (LCA) is a flavonoid derived from licorice root. It possesses various pharmacological properties, including anti-tumor, anti-inflammatory, and antioxidant effects. LCA has the ability to hinder the growth of tumor cells, induce their programmed cell death, and impede the spread of tumors to distant sites. However, its impact on the resistance of glioma cells to TMZ (temozolomide) has not been fully understood.^3^^,^^4^ The activation of the Toll-Like Receptor 4 (TLR4)/Nuclear Factor Kappa B (NF-κB) signaling pathway is strongly associated with the development of several types of cancer, including glioma. This pathway could potentially serve as a target for glioma treatment.^5^^,^^6^ The precise role of LCA in regulating the TLR4/NF-κB signaling pathway, its impact on glioma cell proliferation, and its effect on enhancing TMZ sensitivity have not been thoroughly elucidated. This study aimed to examine the impact of LCA on the growth, spread, and resistance to drugs in glioma cells. Additionally, it evaluated how LCA regulates the TLR4/NF-κB signaling pathway, with the goal of establishing a theoretical foundation for therapeutic treatment.

## Materials and methods

### Materials & reagents

LCA was purchased from Shanghai Tongtian Biological Company (purity ≥98 %); Human glioma cells U251 were purchased from ATCC Company in the United States; TMZ was purchased from Shanghai Enzyme Research Biological Company; CCK-8 reagent and MTT reagent were purchased from Taizhou Yunke Biological Company; The transfection reagent Lipofectamine3000, Transwell chamber and Matrigel were purchased from Beijing Solaibao Technology Co., Ltd.; Bicinchoninic Acid (BCA) and RIPA protein lysate were purchased from Sigma in the United States; Cyclin D1, E-cadherin, N-cadherin, TLR4 and NF-κB monoclonal antibodies were purchased from Santa Cruz in the United States. The GAPDH internal control antibody was purchased from CST in the United States; the Goat anti-rabbit IgG secondary antibody was purchased from Wuhan Doctor Biotech Company; the Trizol reagent was purchased from Invitrogen in the United States; SuperReal Fluorescence Quantitative Premixed Reagent Enhanced (SYBR Green) and Quant cDNA First Strand Synthesis Kit was purchased from Beijing Tiangen Biochemical; Negative Control (si-NC) and TLR4 Oligonucleotide Inhibitor (si-TLR4) were purchased from Guangzhou RiboBio Co., Ltd.; The overexpression plasmid (pcDNA) and TLR4 overexpression plasmid (pcDNA-TLR4) were purchased from Shanghai Jiman Biological Company; Human fibronectin was purchased from Beijing Biolebo Technology Co., Ltd.

### Methods

#### U251 cell culture and U251/TMZ cell induction

U251 cells were cultured with DMEM medium supplemented with 10 % FBS (fetal bovine serum). Incubation conditions: 37 °C, 5 % CO2. U251 cells, respectively, were cultured with different doses of TMZ which were used to set the concentration gradient: TMZ-0, 1, 2, 4, 8, 16, 32, 64 μg/mL.^7^ The cells were stably grown at the lowest concentration for 7 days and then the next concentration treatment, if the U251 cells could grow stably at a concentration of 8 μg/mL, they could be called U251/TMZ cells, and placed in a medium containing 1 μg/mL TMZ to maintain their drug resistance.

#### Experimental processing and grouping

U251 cells and U251/TMZ cells were set as a control group, low-dose group, medium-dose group, and high-dose group, respectively, and the control group was cultured with DMEM medium +10 % FBS, and the three dose groups were cultured with 5, 10, and 20 μmoL/L LCA,^8^ respectively, and the cells were collected after 48 h of culture. Negative Control group (si-NC group), TLR4 oligonucleotide inhibitor group (si-TLR4 group), LCA high dose + overexpression plasmid group (high dose + pcDNA group) and LCA high dose + TLR4 overexpression vehicle group (high dose +pcDNA-TLR4 group) were set up for TLR4/NF-κB signaling pathway. 2 × 10^5^ cells were seeded in 6-well plates, si-NC and si-TLR4 were transfected into U251 cells and U251/TMZ cells respectively by Lipofectamine3000 when the cells were in logarithmic phase. (si-NC group and si-TLR4 group, respectively) PcDNA and pcDNA-TLR4 were transfected into U251 cells and U251/TMZ cells respectively by the above transfection methods. After transfection for 48 h, the cells were collected, and then they were cultured in a medium containing 20 μmoL/L licochlorone A for 48 h (high dose +pcDNA group and high dose +pcDNA-TLR4 group, respectively). Subsequent experiments were conducted after the cells were collected.

#### CCK-8 assay was used to the cell survival rate

(1) To verify the drug resistance of U251/TMZ cells to TMZ: 5 × 103 cells of U251 cells and U251/TMZ cells in logarithmic growth period were inoculated into 96-well plates, treated with TMZ-0, 1, 2, 4, 8, 16, 32 and 64 μg/mL for 48 h, and 10 μL CCK-8 solution was added to each well. After continuous culture for 2 hours, the absorbance (OD 450 nm) of each well was detected by SpectraMax190 light absorption enzyme-labeled instrument (Shanghai Damai Biological Company), and the TMZ IC50 value was calculated by Msvbvm50 software. (2) Inoculate U251 cells and U251/TMZ cells in logarithmic growth phase into 96-well plates (5 × 10^3^ cells/well) respectively, treat them for 48 h according to the experimental method of part 1.2.2, then add 10 μL CCK-8 solution respectively, continue to culture for 2 h, and then detect the absorbance (OD 450 nm) of each well by enzyme-labeled instrument. The cell survival rate was calculated (OD value of different dose groups/control group × 100 % or OD value of si-TLR4 group/OD value of Si-NC group × 100 % or OD value of high dose +pcDNA-TLR4 group/high dose +pcDNA group × 100 %).

#### An adhesion test was used to detect cell adhesion ability

30 μL of human fibronectin (100 mg/mL) was added to 96 well plates, incubated at 37 °C for 1 h, then washed with PBS and dried at room temperature. Each group of U251 cells and U251/TMZ cells from part 1.2.2 were inoculated into the above 96-well plates (4 × 10^4^ cells/well), cultured for 1.5 h, washed with PBS, incubated at 37 °C for 4 h, dissolved with 150 μL DMSO solution, and detected OD 570 nm by enzyme-labeled instrument. The cell adhesion rate was calculated =(ODvalueofdifferentdosegroups/controlgrouporsi−TLR4group/si−NCgrouporhighdose+pcDNA−TLR4group/highdose+pcDNAgroup)×100░%.

#### Transwell experiment was used to detect cell migration and invasion ability

Migration experiment: U251 cells and U251/TMZ cells of each group from part 1.2.2 were diluted with serum-free medium, and 200 μL of cell suspension (1 × 10^5^ cells) was inoculated into the upper chamber of Transwell, and 500 μL of complete medium was added into the lower chamber, incubated at 37 °C for 24 h, wiped off the remaining cells in the upper chamber, fixed the cells on the lower chamber with methanol for 30 min, and stained with crystal violet for 20 min. Invasion experiment: Transwell was precoated with Matrigel glue diluent and left for 5 h, and the subsequent experimental steps were the same as the migration experiment.

#### Real-time fluorescence quantitative polymerase chain reaction (qRT-PCR) was used to detect the expression levels of TLR4 and NF-κB mRNA in cells

Take U251 cells and U251/TMZ cells of each group from part 1.2.2, extract total RNA from cells with Trizol reagent and detect RNA concentration, and take 2 μL RNA for reverse transcription experiment to synthesize cDNA. QRT-PCR reaction with cDNA as a template: SYBR Green Master Mix 10 μL, forward primer 0.8 μL, Cdna 1 μL, ddH2O to make up the system to 20 μL. Reaction conditions: 95 °C for 2 min, 95 °C 95 °C 15 s, 60 °C for 30 s, 72 °C for 30 s, with 40 cycles. The relative expressions of TLR4 and NF-κB mRNA were calculated by the 2-^ΔΔCt^ method with GAPDH as an internal reference. TLR4 forward primer 5′-CTGTCCCATCGATACGC-3′, reverse primer 5′-CTTGCCTCAAGTCCTTG-3′; NF-κB forward primer 5′-CACCCTGACCCTTGCCTAT-3′, reverse primer 5′-CCCACACTACCATTT-3′; GAPDH forward primer 5′-CACTACCGTACTCGACCACCA-3′ and reverse primer 5′-ATGTCGTTCCCCACCACCT-3′ were purchased from Shanghai Bioengineering Company.

#### Western blot was used to detect the expression of TLR4, NF-κB, CyclinD1, E-cadherin and N-cadherin proteins

Take U251 cells and U251/TMZ cells of each group from part 1.2.2, add RIPA lysate to extract the total protein, determine the concentration by BCA, separate the protein by SDS-PAGE reaction, and transfer it to PVDF membrane, and seal the membrane with 5 % skim milk for 2 hours. After washing, they were put into GAPDH antibody containing TLR4 (dilution ratio 1:800), NF-κB (dilution ratio 1:800), CyclinD1 (dilution ratio 1:1000), E-cadherin (dilution ratio 1:1000) and N-cadherin (dilution ratio 1:1000) respectively. After washing the membrane, they were incubated in the diluent containing secondary antibody (dilution ratio 1:5000) for 1 h, exposed and developed in a dark room, and the gray value of each band was analyzed by ImageJ software. The relative expression of each protein was calculated with GAPDH as an internal reference.

### Statistical analysis

Statistical software SPSS 26.0 was used to analyze the data, and the measurement data were all in accordance with the normal distribution and represented by (χ ± *s*). Independent sample *t-*test was used for comparison between the two groups, one-way ANOVA was used for comparison between multiple groups, and LSD-t-test was used for comparison between pairwise groups, with a difference of *p <* 0.05 being statistically significant.

## Results

### Verification of drug resistance of U251/TMZ cells

The results of CCK-8 experiment showed that after TMZ intervention, compared with U251 cells, the survival rate and IC50 of U251/TMZ cells increased significantly (*p* < 0.05), as shown in Table 1. The results showed that U251/TMZ cells were resistant to TMZ.

**Table 1:** U251/TMZ validation of cell resistance (χ ± *s, n* = 9).

**Group**	**Cell viability**								**IC50 (μg/mL)**
	**TMZ-0 μg/mL**	**TMZ-1 μg/mL**	**TMZ-2 μg/mL**	**TMZ-4 μg/mL**	**TMZ-8 μg/mL**	**TMZ-16 μg/mL**	**TMZ-32 μg/mL**	**TMZ-64 μg/mL**	
U251 cells	100.00±0.01	96.33±1.25	55.46±2.48	45.19±3.06	42.11±3.05	40.03±2.85	38.45±2.11	30.26±3.02	2.66±0.28
U251/TMZ cells	100.00±0.03	97.41±1.33	82.16±13.38	76.31±5.43	70.03±5.02	51.13±5.04	43.16±4.38	40.08±3.16	27.41±2.16
*t*	0.000	1.775	5.886	14.979	14.260	5.751	2.906	6.740	34.090
p	1.000	0.095	0.000	0.000	0.000	0.000	0.010	0.000	0.000

### Effect of LCA on the biological behavior of U251 cells and U251/TMZ cells

**Fig. 1:**
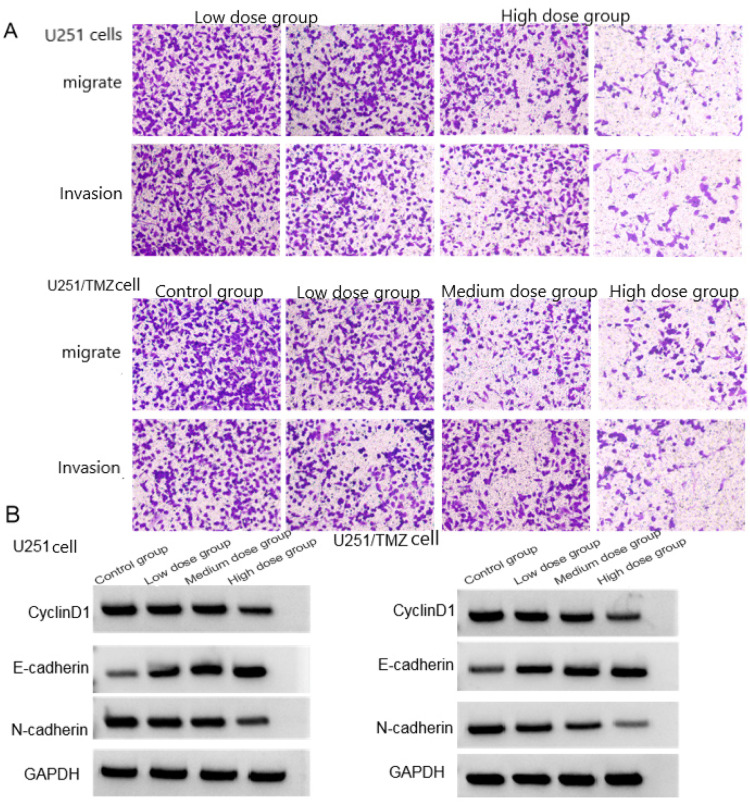
Effects of LCA to U251 cells and U251/TMZ cells on cell migration and invasion. (A) Migration and invasion graph; (B) Protein graph.

The survival rate, cell adhesion rate, number of migrating cells and number of invading cells of U251 cells and U251/TMZ cells in high-dose group were lower than those in middle-dose group < low-dose group < control group (*p* < 0.05), and the levels of CyclinD1 and N-cadherin protein in U251 cells and U251/TMZ cells in high-dose group were lower than those in middle-dose group < low-dose group < control group, high-dose (*p* < 0.05), see Fig. 1, Table 2, Table 3.

**Table 2:** Effects of LCA on the biological behavior of U251 cells (χ ± *s, n* = 9).

Group	Cell viability (%)	Cell adhesion rate (%)	Number of migrating cells	Number of invading cells	CyclinD1	E-cadherin	N-cadherin
Control group	100.01±0.05	82.51±7.54	245.16±41.72	253.26±44.42	0.85±0.11	0.28±0.05	0.82±0.06
Low-dose group	75.24±15.08^a^	70.03±5.34^a^	200.32±40.67^a^	201.38±32.26^a^	0.63±0.15^a^	0.42±0.08^a^	0.68±0.10^a^
Medium-dose group	52.02±5.34^a^^,^^b^	55.41±8.47^a^^,^^b^	142.16±37.14^a^^,^^b^	155.94±26.38^a^^,^^b^	0.50±0.08^a^^,^^b^	0.65±0.11^a^^,^^b^	0.52±0.07^a^^,^^b^
High-dose group	32.28±6.76^a^^,^^b^^,^^c^	30.92±4.36^a^^,^^b^^,^^c^	62.31±10.77^a^^,^^b^^,^^c^	66.75±9.27^a^^,^^b^^,^^c^	0.34±0.09^a^^,^^b^^,^^c^	0.80±0.16^a^^,^^b^^,^^c^	0.40±0.05^a^^,^^b^^,^^c^
*F*	102.231	100.412	45.926	59.352	34.069	41.633	57.771
p	0.000	0.000	0.000	0.000	0.000	0.000	0.000

Compared with the control group

a *p* < 0.05; Compared to the low-dose group

b *p* < 0.05; Compared with the mid-dose group.

c *p* < 0.05.

**Table 3:** Effects of LCA on the biological behavior of U251/TMZ cells (χ ± *s, n* = 9).

Group	Cell viability (%)	Cell adhesion rate (%)	Number of migrating cells	Number of invading cells	CyclinD1	E-cadherin	N-cadherin
Control group	100.03±0.06	83.26±8.22	263.21±40.03	244.74±40.03	0.82±0.10	0.29±0.04	0.83±0.05
Low-dose group	72.85±16.30^a^	71.02±5.94^a^	196.38±45.21^a^	198.24±36.95^a^	0.70±0.06^a^	0.44±0.06^a^	0.71±0.08^a^
Medium-dose group	50.27±6.28^a^^,^^b^	52.18±7.10^a^^,^^b^	132.28±33.62^a^^,^^b^	148.92±22.63^a^^,^^b^	0.54±0.09^a^^,^^b^	0.68±0.07^a^^,^^b^	0.50±0.07^a^^,^^b^
High-dose group	35.29±6.02^a^^,^^b^^,^^c^	33.19±3.75^a^^,^^b^^,^^c^	60.09±8.92^a^^,^^b^^,^^c^	58.74±5.16^a^^,^^b^^,^^c^	0.30±0.05^a^^,^^b^^,^^c^	0.81±0.13^a^^,^^b^^,^^c^	0.33±0.04^a^^,^^b^^,^^c^
*F*	83.936	103.442	50.069	64.993	75.174	72.933	115.071
p	0.000	0.000	0.000	0.000	0.000	0.000	0.000

Compared with the control group.

a *p* < 0.05; Compared to the low-dose group.

b *p* < 0.05; Compared with the mid-dose group.

c *p* < 0.05.

### Effect of LCA on TLR4/NF-κB signaling pathway

**Fig. 2:**
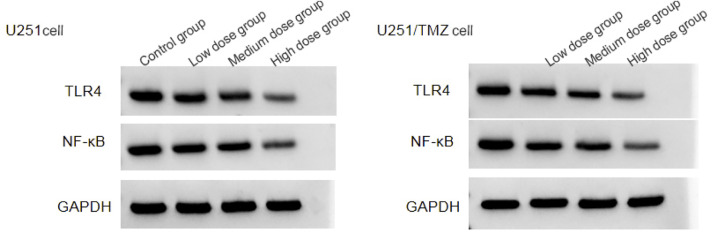
Protein expression levels of TLR4/NF-κB signaling pathway.

The mRNA levels of TLR4 and NF-κB in U251 cells and U251/TMZ cells in high dose group were lower than those in middle dose group < low dose group < control group (*p* < 0.05), and the protein levels of TLR4 and NF-κB in U251 cells and U251/TMZ cells in high dose group were lower than those in middle dose group < low dose group < control group (*p* < 0.05), see Fig. 2, Table 4, Table 5.

**Table 4:** Effects of LCA on TLR4/NF-κB signaling pathway (χ ± *s, n* = 9).

**Group**	**U251cells**				**U251/TMZ cells**			
	**TLR4 mRNA**	**NF-κB mRNA**	**TLR4 protein**	**NF-κB protein**	**TLR4 mRNA**	**NF-κB mRNA**	**TLR4 protein**	**NF-κB protein**
Control group	1.00±0.02	1.00±0.05	0.88±0.19	0.96±0.15	1.00±0.04	1.00±0.03	0.87±0.15	0.92±0.14
Low-dose group	0.71±0.16^a^	0.73±0.10^a^	0.62±0.11^a^	0.70±0.11^a^	0.80±0.10^a^	0.77±0.09^a^	0.59±0.10^a^	0.68±0.18^a^
Medium-dose group	0.50±0.11^a^^,^^b^	0.52±0.09^a^^,^^b^	0.42±0.08^a^^,^^b^	0.53±0.12^a^^,^^b^	0.60±0.08^a^^,^^b^	0.55±0.10^a^^,^^b^	0.39±0.08^a^^,^^b^	0.40±0.10^a^^,^^b^
High-dose group	0.31±0.08^a^^,^^b^^,^^c^	0.34±0.07^a^^,^^b^^,^^c^	0.30±0.05^a^^,^^b^^,^^c^	0.40±0.09^a^^,^^b^^,^^c^	0.41±0.05^a^^,^^b^^,^^c^	0.38±0.11^a^^,^^b^^,^^c^	0.21±0.04^a^^,^^b^^,^^c^	0.25±0.05^a^^,^^b^^,^^c^
*F*	70.813	113.824	40.581	36.877	113.605	83.846	71.200	49.428
p	0.000	0.000	0.000	0.000	0.000	0.000	0.000	0.000

Compared with the control group.

a *p* < 0.05; Compared to the low-dose group.

b *p* < 0.05; Compared with the mid-dose group.

c *p* < 0.05.

**Table 5:** Effect of silencing TLR4/NF-κB signaling pathway on the biological behavior of U251 cells (χ ± *s, n* = 9).

Group	Cell viability (%)	Cell adhesion rate (%)	Number of migrating cells	Number of invading cells	CyclinD1	E-cadherin	N-cadherin	TLR4 protein	NF-κB protein
si-NC group	100.00±0.05	83.12±8.54	271.45±30.02	265.32±31.85	0.84±0.11	0.31±0.08	0.85±0.08	0.90±0.15	0.93±0.18
si-TLR4 group	68.54±12.84	66.29±12.09	152.29±30.76	147.19±26.38	0.55±0.13	0.69±0.15	0.62±0.16	0.22±0.05	0.28±0.06
*t*	7.350	3.411	8.317	8.768	5.109	6.706	3.857	12.902	10.277
p	0.000	0.004	0.000	0.000	0.000	0.000	0.001	0.000	0.000

### Effects of silencing TLR4/NF-κB signaling pathway on biological behavior of U251 cells and U251/TMZ cells

**Fig. 3:**
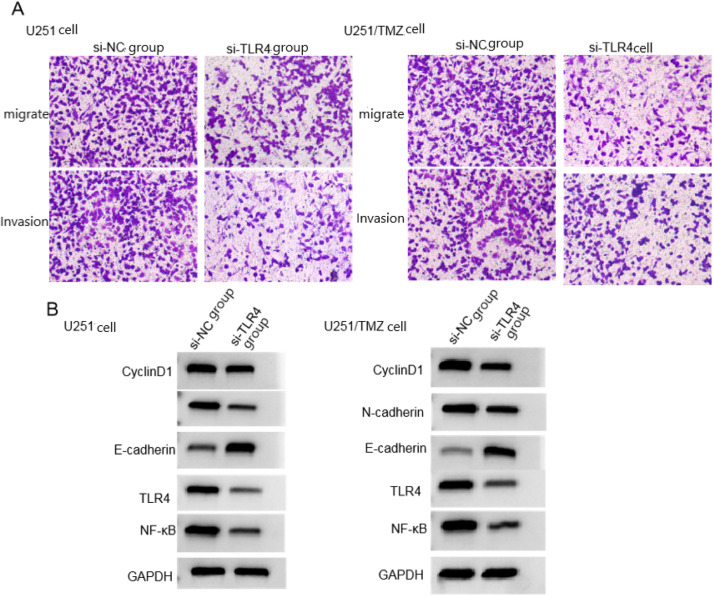
Effects of silencing TLR4/NF-κB signaling pathway on cell migration, invasion and related protein expression of U251 cells and U251/TMZ cells. (A) Migration and invasion graph; (B) Protein graph.

The survival rate and cell adhesion rate of U251 cells and U251/TMZ cells in si-TLR4 group were lower than those in si-NC group, and the number of migrating cells and invading cells was less than those in the si-NC group. The protein levels of CyclinD1, N-cadherin, TLR4, and NF-κB were lower than those in si-NC group, and the protein level of E-cadherin was higher than that in si-NC group (*p* < 0.05), see Fig. 3, Table 5, Table 6.

**Table 6:** Effect of silencing TLR4/NF-κB signaling pathway on the biological behavior of U251/TMZ cells (χ ± *s, n* = 9).

Group	Cell viability (%)	Cell adhesion rate (%)	Number of migrating cells	Number of invading cells	CyclinD1	E-cadherin	N-cadherin	TLR4 protein	NF-κB protein
si-NC组	100.85±0.07	84.55±9.65	277.16±36.29	259.31±32.01	0.86±0.13	0.33±0.09	0.88±0.15	0.94±0.17	0.90±0.15
si-TLR4组	70.14±13.25	68.74±13.06	163.29±25.16	158.94±30.08	0.64±0.18	0.67±0.12	0.58±0.17	0.18±0.02	0.24±0.05
*t*	6.953	2.921	7.736	6.855	2.972	6.800	3.970	13.320	12.523
p	0.000	0.010	0.000	0.000	0.009	0.000	0.001	0.000	0.000

### Effects of TLR4/NF-κB signal pathway activation combined with LCA on biological behavior of U251 cells and U251/TMZ cells

**Fig. 4:**
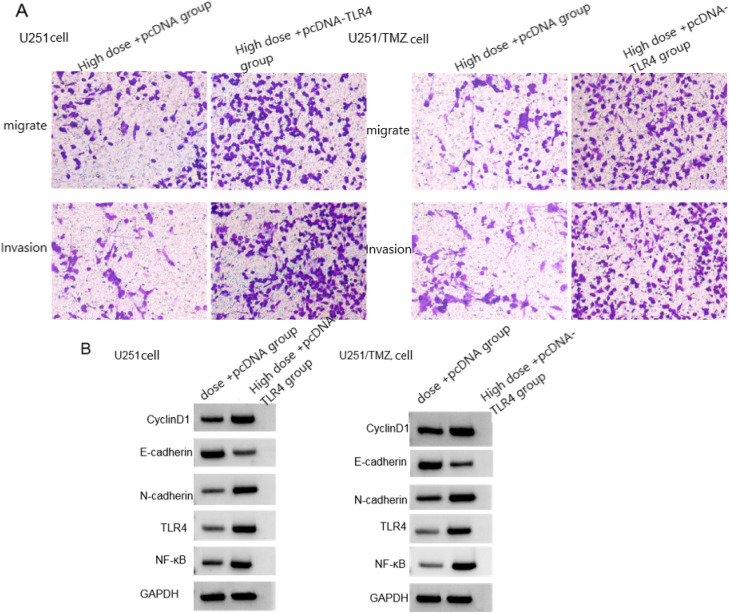
Effects of TLR4/NF-κB signaling pathway activation combined with LCA on migration, invasion and related protein expression in U251 cells and U251/TMZ cells. (A) Migration and invasion graph; (B) Protein graph.

Compared with the high-dose +pcDNA group, the survival rate and cell adhesion rate of U251 cells and U251/TMZ cells in the high-dose +pcDNA-TLR4 group increased, the number of migrating cells and invading cells increased, the protein levels of CyclinD1, N-cadherin, TLR4 and NF-κB increased, and the protein level of E-cadherin decreased (*p* < 0.05), see Fig. 4, Table 7, Table 8.

**Table 7:** Effects of TLR4/NF-κB signaling pathway activation combined with LCA on the biological behavior of U251 cells (χ ± *s, n* = 9).

Group	Cell viability (%)	Cell adhesion rate (%)	Number of migrating cells	Number of invading cells	CyclinD1	E-cadherin	N-cadherin	TLR4 protein	NF-κB protein
High-dose +pcDNA group	36.22±5.28	32.49±3.55	61.29±9.25	60.03±6.28	0.33±0.08	0.79±0.20	0.32±0.09	0.29±0.05	0.39±0.11
High-dose +pcDNA-TLR4 group	70.02±13.28	62.39±15.28	128.64±32.88	139.67±36.55	0.68±0.18	0.49±0.12	0.78±0.20	0.88±0.19	0.91±0.20
*t*	7.095	5.718	5.915	6.442	5.331	3.859	6.292	9.009	6.834
p	0.000	0.000	0.000	0.000	0.000	0.001	0.000	0.000	0.000

**Table 8:** Effects of TLR4/NF-κB signaling pathway activation combined with LCA on the biological behavior of U251/TMZ cells (χ ± *s, n* = 9).

Group	Cell viability (%)	Cell adhesion rate (%)	Number of migrating cells	Number of invading cells	CyclinD1	E-cadherin	N-cadherin	TLR4 protein	NF-κB protein
High-dose +pcDNA group	37.88±10.62	33.95±10.32	62.41±15.28	63.19±18.21	0.31±0.05	0.82±0.21	0.36±0.10	0.19±0.04	0.22±0.05
High-dose +pcDNA-TLR4 group	72.41±18.32	68.57±15.25	145.29±35.26	168.42±36.15	0.73±0.20	0.35±0.10	0.84±0.22	0.93±0.28	0.97±0.25
*t*	4.892	5.640	6.470	7.799	6.112	6.062	5.959	7.849	8.825
p	0.000	0.000	0.000	0.000	0.000	0.000	0.000	0.000	0.000

## Discussion

The development of glioma may be attributed to angiogenesis, activation of oncogenes, and inactivation of tumor suppressor genes. The emergence of TMZ resistance may be associated with the proliferation and metastasis of cancer cells, leading to an increased likelihood of glioma recurrence and impacting patient prognosis.^9^^,^^10^ Hence, investigating the drug resistance of TMZ in glioma cells can enhance the prognosis of patients.

LCA suppresses the growth of tumor cells, triggers programmed cell death, halts the progression of the cell cycle, hinders the phosphorylation of the Phosphatidylinositol-3-Kinase (PI3 K)/Protein Kinase B (AKT) signaling pathway, and diminishes the amounts of proteins that prevent cell death and promote cell proliferation.^11^^,^^12^ Tseng et al.^13^ demonstrated that LCA has the ability to suppress the production of LC3 and impair the growth and spread of cancer cells. The study findings demonstrated a negative correlation between the dosage of LCA and cell survival, cell adhesion rate, migration, and invasion capacity. Wang Jiu and colleagues demonstrated in their study^14^ that LCA has the ability to suppress the growth of glioma cells and trigger their programmed cell death. Mu et al.^15^ have shown that LCA has the ability to control the expression of the Rab23 gene and reduce the proliferation capacity of U251 cells, thereby confirming the findings of this investigation. Epithelial-Mesenchymal Transition (EMT) is linked to the movement and infiltration of tumors. An elevated EMT level in tumor tissue suggests an enhanced ability for tumor metastasis. This increase in EMT level corresponds to a down-regulation of E-cadherin expression and an up-regulation of N-cadherin expression. These changes facilitate the invasion and metastasis of cells to neighboring tissues. CyclinD1 is a biomarker of cell cycle progression that stimulates cell proliferation and plays a crucial role in the transition from the G1 phase to the S phase of the cell cycle. The study's findings demonstrated that LCA has the ability to enhance the expression of E-cadherin while suppressing the expression of CyclinD1 and N-cadherin. This confirms that LCA may effectively hinder the process of Epithelial-Mesenchymal Transition (EMT) in glioma cells, disrupt the histological barrier of tumor cells, and diminish their capacity for migration and invasion. Glioma has the ability to cause a halt in the cell cycle, decrease the expression of CyclinD1, and hence hinder the growth of glioma cells. Han et al.^16^ demonstrated that LCA suppressed c-Met signaling and increased the responsiveness of non-small cell lung cancer cells to gefitinib. According to Wu et al.,^17^ they demonstrated that LCA has the ability to decrease the expression of ABCG2 and increase the responsiveness of cancer cells that are resistant to multiple treatments with chemotherapy medications. Nevertheless, the impact of LCA on TMZ resistance remains unclear. This study, however, discovered that as the dosage of LCA increased, the proliferation, adhesion, migration, and invasion capabilities of U251/TMZ cells were notably diminished. Additionally, the expression of CyclinD1 and N-cadherin was down-regulated, while the expression of E-cadherin was up-regulated. These findings suggest that LCA can mitigate TMZ resistance by suppressing cell proliferation, migration, and invasion.

TLR4 is primarily found in immune cells, including T/B lymphocytes and macrophages. It can also be present in non-immune cells, such as tumor cells. The presence of TLR4 in tumor cells can stimulate tumor development and facilitate the evasion of the immune system by the tumor. TLR4 triggers the activation of the NF-κB gene, which in turn facilitates inflammatory and immunological responses. Additionally, TLR4 activation leads to the activation of CyclinD1 and stimulates the involvement of cells in the process of tumor formation and progression during the G1/S phase.^18^^,^^19^ Kına et al.^20^ discovered that the activation of the TLR4/NF-κB signaling pathway might stimulate the progression of glioma. This study aimed to observe the impact of silencing the TLR4/NF-κB signaling pathway on the biological behavior of U251 cells. The findings revealed that inhibiting the TLR4/NF-κB signaling pathway could effectively suppress cell proliferation and adhesion, as well as attenuate cell migration and invasion capabilities. The study conducted by Liu et al.^21^ demonstrated that the suppression of TLR4 can impede the production of NF-κB and decrease the proliferative capacity of glioma cells. These findings provide further support for the results drawn from this investigation. Liu et al.^22^ demonstrated that the TLR4/NF-κB signaling pathway is activated. et al.^23^ shows that the activation of the TLR4/NF-κB signaling pathway is linked to chemotherapy resistance in gastric cancer cells. The study discovered that inhibiting the TLR4/NF-κB signaling pathway reduced the growth and spread of U251/TMZ cells, resembling the findings of the previous study. To investigate the relationship between LCA and TLR4/NF-κB signaling pathways in glioma, U251 cells and U251/TMZ cells were simultaneously exposed to LCA and pcDNA-TLR4. The findings revealed a notable increase in cell survival rate and cell adhesion rate, as well as a significant rise in the number of migrating cells and invasive cells. These results suggest that the activation of the TLR4/NF-κB signaling pathway may contribute to TMZ resistance. Furthermore, LCA was found to inhibit the activation of the TLR4/NF-κB signaling pathway, thereby reversing TMZ drug resistance. This discovery highlights the potential of targeting the TLR4/NF-κB signaling pathway as a therapeutic approach for glioma.

To summarize, LCA has the ability to suppress the growth, movement, and invasion of glioma cells. It can also counteract resistance to TMZ drugs. The mechanism by which it achieves these effects may involve inhibiting the activation of the TLR4/NF-κB signaling pathway. This discovery opens up new possibilities for identifying clinical drugs to treat glioma patients who are resistant to TMZ. Nevertheless, the investigation of LCA, the TLR4/NF-κB signaling pathway, and TMZ resistance in gliomas is currently in the preliminary phase. In order to validate the efficacy of their therapeutic application, additional in vivo experiments and clinical trials are required.

## Declaration of competing interest

The authors declare no conflicts of interest.
